# Microbial Communities of Conducting and Respiratory Zones of Lung-Transplanted Patients

**DOI:** 10.3389/fmicb.2016.01749

**Published:** 2016-11-07

**Authors:** Marie Beaume, Vladimir Lazarevic, Thilo Köhler, Nadia Gaïa, Oriol Manuel, John-David Aubert, Loïc Baerlocher, Laurent Farinelli, Paola Gasche, Jacques Schrenzel, Christian van Delden, the Swiss Transplant Cohort Study

**Affiliations:** ^1^Transplant Infectious Diseases Unit, Geneva University Hospitals and Department of Microbiology and Molecular Medicine, University of GenevaGeneva, Switzerland; ^2^Genomic Research Laboratory, Geneva University HospitalsGeneva, Switzerland; ^3^Infectious Diseases Service and Transplantation Center, Lausanne University Hospital CenterLausanne, Switzerland; ^4^Division of Pulmonary Diseases, Lausanne University Hospital CenterLausanne, Switzerland; ^5^Fasteris SA, Plan-les-OuatesSwitzerland; ^6^Division of Pulmonary Diseases, Geneva University HospitalsGeneva, Switzerland

**Keywords:** microbiota, lung transplantation, conducting airways, respiratory airways, lung allograft

## Abstract

**Background:** Lung transplantation (LT) is a recognized treatment for end-stage pulmonary disease. Bacteria from the recipient nasopharynx seed the new lungs leading to infections and allograft damage. Understanding the characteristics and topological variations of the microbiota may be important to apprehend the pathophysiology of allograft dysfunction.

**Objectives:** To examine the characteristics and relationship of bacterial compositions between conducting and respiratory zones of the allograft.

**Methods:** We performed 16S rRNA gene sequencing on bronchial aspirates (BAs) and bronchoalveolar lavages (BALs) collected in pairs in 19 patients at several time-points post-LT.

**Results:** The respiratory zone was characterized independently of the time post-LT by a higher bacterial richness than the conducting zone (*p* = 0.041). The phyla Firmicutes and Proteobacteria dominated both sampling zones, with an inverse correlation between these two phyla (Spearman *r* = –0.830). Samples of the same pair, as well as pairs from the same individual clustered together (Pseudo-*F* = 3.8652, *p* < 0.01). Microbiota of BA and BAL were more closely related in samples from the same patient than each sample type across different patients, with variation in community structure being mainly inter-individual (*p* < 0.01). Both number of antibiotics administered (*p* < 0.01) and time interval post-LT (*p* < 0.01) contributed to the variation in global microbiota structure. Longitudinal analysis of BA–BAL pairs of two patients showed dynamic wave like fluctuations of the microbiota.

**Conclusions:** Our results show that post-transplant respiratory zones harbor higher bacterial richness, but overall similar bacterial profiles as compared to conductive zones. They further support an individual microbial signature following LT.

## Introduction

Lung transplantation (LT) is the sole therapeutic intervention for patients with end-stage pulmonary disease. With 3893 lungs transplanted in 2013 in 242 European and US lung transplant centers, this procedure is an increasing common approach according to the International Society for Heart and LT Registry (Yusen et al., 2015). However, the outcome after LT is worse compared to other solid organ transplantations, with a median survival of 5.7 years post-LT (Yusen et al., 2015). Mortality during the 1st year post-LT is mainly due to infections, whereas late mortality is linked to chronic rejection, notably the bronchiolitis obliterans syndrome (BOS). Cystic fibrosis (CF) lung-transplant recipients are at particularly high risk of infection due to chronic pre-transplant colonization with microorganisms including *Staphylococcus aureus, Haemophilus influenzae*, and *Pseudomonas aeruginosa*, the latter being the most frequent pathogen in adult CF-patients (Cystic Fibrosis Foundation, 2011). Preventing infections is one of the major challenges to increase allograft survival and to improve outcome after LT.

**FIGURE 1:**
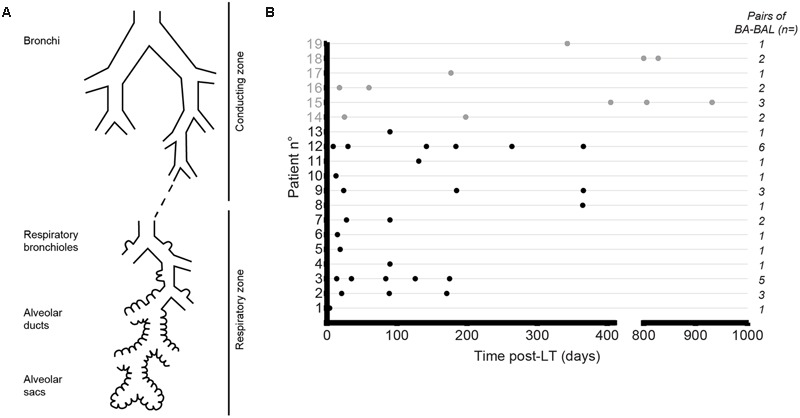
**Collection of bronchial aspirate–bronchoalveolar lavage (BA–BAL) pairs from lung-transplant recipients. (A)** Both zones were explored during the same procedure, with the conducting zone being sampled by performing a BA and the respiratory zone by BAL. **(B)** Dots represent BA–BAL pairs collected for each of the 19 patients after lung-transplantation. Gray dots refer to CF patients, whereas black dots depict non-CF patients.

Airways are composed of several interconnected compartments, i.e., the sinuses, the trachea, the conducting, and the respiratory zones (**Figure 1A**). After removal of the major site of infections (i.e., the lungs) by LT, bacteria that remain in the sinuses seed the allograft. Polymicrobial communities of LT-recipients appear to be distinct from those of healthy subjects (Charlson et al., 2012; Borewicz et al., 2013). Relationships between upper and lower airways microbiota are not fully understood. Our previous study demonstrated that colonization of the new lungs by *P. aeruginosa* occurs within the 1st days after LT in CF patients (Beaume et al., 2015). Other studies have shown differences between sputum and lower airway microbiota in patients with lung disease such as CF and chronic obstructive pulmonary disease (COPD) (Cabrera-Rubio et al., 2012; Goddard et al., 2012). Despite an increasing number of studies related to the microbiota of LT-recipients, no comparison between the intermediate (conducting zone) and lower (respiratory zone) airway environments has been performed. It is still unknown if transplanted-lungs have a uniform microbiota, or if specific microbial populations colonize different regions of the allograft. While the presence of the mucociliary movements can favor the homogeneity of the microbiota in the airways, recurrent bacterial seeding of the graft from the naso and oro-pharyngeal reservoir may establish a gradient from the upper to the lower airways. Understanding the regional disparities of the airway microbiota may therefore contribute to characterize the complex graft environment and to apprehend the pathophysiology of graft infections.

The objective of this study was to characterize and compare the microbiota from bronchial aspirates (BAs) and bronchoalveolar lavages (BALs) samples collected simultaneously from CF and non-CF lung-transplant recipients. We thus assessed the microbiota of the conducting and respiratory zones and, the impact of time post-transplantation, CF, use of antibiotics and patient age on microbiota profiles. We further analyzed changes of the allograft microbiota in sequential airway samples from two non-CF patients.

## Materials and Methods

### Subject Recruitment and Sample Collection

This prospective observational work was part of the Swiss Transplant Cohort Study (STCS) (Koller et al., 2013). Authorization to use all clinical samples for research purposes was obtained from the local ethical committee and all patients provided their written consent. Pulmonary function, including the forced expiratory volume (FEV_1_), was followed by routine spirometry at regular time intervals after LT. The clinical diagnosis of BOS was defined by a sustained pulmonary decline, with a FEV_1_ reduction of >20% compared to baseline, after excluding confounding factors (Verleden et al., 2014). The two best FEV_1_ values at a > 3 week time interval obtained during the first 6 months after LT were used to define the baseline, for the purpose of this study.

Pairs of BAs and BALs were collected during routine surveillance bronchoscopies. BAs consisted of respiratory secretions aspirated below the bronchial suture, and representing heterogeneous samples from both lungs at the level of the conducting zone. After rinsing the working channel of the bronchoscope with 5 mL saline, the bronchoscope was wedged in 3–5th generation airways and washed again by the instillation of 150 ml of NaCl 0.9% into the distal airways. BAL fluid was obtained by aspiration of 10–20 ml distal respiratory secretions, thus representing a more homogenous sample from a distal respiratory zone. 1 mL of each airway sample was immediately stored at -80°C after sampling.

### Evaluation of Potential Cross-Contamination between BA and BAL

Ten milliliters of a solution containing 8.5 × 10^3^ cells/mL of *P. aeruginosa* strain PAO1 were aspirated *ex vivo* with the bronchoscope. This first aspirate was called “pseudo-BA.” After rinsing the working channel of the bronchoscope with 5 mL NaCl 0.9%, 150 mL of NaCl were instilled through the bronchoscope in a solution containing 1.9 × 10^5^ cells of *S. aureus* strain Newman. 10 ml of this mixture was then aspirated using the same bronchoscope in a sterile container. This second aspirate was called “pseudo-BAL.” Hundred microliter of the pseudo-BA and pseudo-BAL were plated in duplicate on Cetrimide Agar and LB supplemented with 10 μg/mL of Aztreonam. Plates were incubated 24 h at 37°C. Bacterial DNA was also extracted from 1 mL of the pseudo-BA and pseudo-BAL, and submitted to analysis by qPCR as described in the Supplementary Material.

### gDNA Extraction

For 16S analysis, genomic DNA was isolated from BA and BAL using the DNeasy kit (QIAGEN) according to the manufacturer’s instructions. Additional details are provided in the Supplementary Material.

### 16S rRNA Gene Amplification, Sequencing, and Taxonomic Analysis

The hypervariable regions V4–V6 of the 16S rRNA gene were amplified by PCR using primers Uni16_518F (CCAGCAGCYGCGGTAAN) and Uni16_1064R (CGACRRCCATGCANCACCT) (Filkins et al., 2012). Additional details are provided in the Supplementary Material.

PCR products were sequenced by using an Illumina MiSeq platform (Fasteris SA, Plan-les-Ouates, Switzerland). We used a paired-end protocol allowing to sequence 300 bases of the 5′-end and 300 bases of the 3′-end of each fragment. Sequence files were deposited in EMBL-ENA under the accession number PRJEB13210. A custom bioinformatic pipeline performed quality filtering to remove low quality reads. The reads obtained were first mapped on the 18S human rRNA gene by using the BWA software (v0.5.9) to deplete contaminant reads. BWA-MEM (Version: 0.7.5a-r405) was then used to map remaining reads onto the Greengenes database (file gg_97_otus_4feb2011) (DeSantis et al., 2006) with 97% identity to assign them to specific taxon. Counts were then normalized as relative frequencies per sample and per taxon.

### Statistical Analysis

Alpha-diversity (non-parametric Shannon index) and richness (S observed) were calculated by using the VEGAN package^1^. Calculation was performed for each sample on 7,000 reads taken randomly from the pool of reads. Variations in the alpha-diversity among samples were tested with a linear regression model with mixed effects because measures were repeated overtime.

To compare bacterial communities, we constructed a Bray–Curtis (Bray and Curtis, 1957) similarity matrix based on the square-root-transformed relative abundance of operational taxonomic units (OTUs). Principal coordinates analysis (PCoA) of Bray–Curtis similarities was performed in PRIMER (Primer-E Ltd., Plymouth, UK). To assess differences between BAs and BALs or CF and non-CF samples, we used one-way permutational multivariate analysis of variance (PERMANOVA) of the Bray–Curtis similarity matrix. Distance-based linear model (DISTLM) was used for analyzing the relationship between bacterial community structures and quantitative continuous variables (age, number of antibiotics used, and day post-LT). Similarity percentage (SIMPER) was used to identify OTUs which contribute to the distinction between BA and BAL samples. In addition, Wilcoxon signed rank test was used to assess statistical significance of differences in taxa abundance between BAs and BALs. *P*-values were adjusted using the false discovery rate (FDR) method for multiple comparisons.

## Results

### Sample and Patient Characteristics

Thirty-eight paired BA and BAL samples were obtained from 19 lung-transplant recipients at different time-points post-LT (**Table 1**; **Figure 1B**). Fifty percent of the BA–BAL pairs were collected during the first 100 days. Thirteen patients were transplanted for COPD, interstitial lung disease or bronchiectasis, whereas six patients were transplanted for end-stage CF-disease. Most patients (89%) underwent bilateral transplantation. Bronchoscopies were performed as part of routine surveillance procedures at regular time intervals during the first 3 years post-LT. Patients #4, #18, and #19 developed a BOS at 91, 794, and 325 days post-LT, respectively. Patients #4 and #19 died during our study at 365 and 424 days post-LT, respectively, from irreversible allograft dysfunction. All patients were on immunosuppressive and antimicrobial treatments according to local practice for LT-recipient management.

**Table 1:** Patient characteristics.

Patient *n*°	Pre-transplant disease^∗^	Gender	Age at LT (years)	Extubation (day post-LT)	CFTR genotype	Transplant type^∗∗^	*P. aeruginosa* colonization before LT
1	COPD	F	63	1		R	No
2	COPD	F	61	41		Bi	No
3	COPD	M	63	0		Bi	No
4	COPD	F	60	2		Bi	No
5	ILD	M	52	34		Bi	Yes
6	ILD	F	44	24		Bi	No
7	COPD	F	55	1		Bi	Yes
8	COPD	F	54	2		Bi	No
9	Br	F	47	50		Bi	Yes
10	Br	F	61	39		Bi	Yes
11	COPD	F	54	1		Bi	No
12	COPD	F	58	1		Bi	No
13	ILD	F	50	41		Bi	No
14	CF	F	32	4	dF508	Bi	Yes
15	CF	M	39	10	nd	Bi	Yes
16	CF	M	15	3	dF508	Bi	Yes
17	CF	M	27	115^∗∗∗^	dF508	(Bi), R	Yes
18	CF	M	23	1	dF508	Bi	Yes
19	CF	M	20	0	dF508	Bi	Yes

*^∗^CF, cystic fibrosis; COPD, chronic obstructive pulmonary disease; ILD, interstitial lung disease; Br, Bronchiectasis. ^∗∗^Bi, bilateral; R, unilateral right. Note that patient #17 was subjected to a bilateral LT 1 year before this study followed by a unilateral right LT, which is the transplantation considered in this study. ^∗∗∗^Removal of tracheostomy tube. nd, not determined.*

### Evaluation of Potential Cross-Contamination between BA and BAL

To evaluate if a cross-contamination could occur between BA and BAL samples, we performed an *ex vivo* control experiment closely mimicking the bronchoscopy procedure. Using the same bronchoscope, we successively aspirated a pure culture of *P. aeruginosa* (pseudo-BA), rinsed the bronchoscope and aspirated a pure culture of *S. aureus* (pseudo-BAL). Whereas the “pseudo-BA” yielded an average of 1.9 × 10^2^ CFU/mL (*SD* = 99.0) *P. aeruginosa* and no *S. aureus*, the “pseudo-BAL” yielded 1.2 × 10^2^ CFU/mL (*SD* = 40.3) of *S. aureus* but no *P. aeruginosa*. The absence of detectable cross-contamination was further confirmed by qPCR as *C*_t_ values obtained in the “pseudo-BAL” for *P. aeruginosa* were similar to those obtained with the no template controls.

### Sequence Data Set

After DNA extraction from native samples (BAs and BALs), the V4–V6 segment of the bacterial 16S rRNA gene was amplified and sequenced using Illumina sequencing technology. A median of 64765 (range 1378–746584) and 57228 (range 3747–1235360) sequences were obtained for the BA and BAL samples, respectively (Supplementary Table S1). Due to low sequencing coverage, the two BA–BAL pairs from patient #18 were not included in the analyses. After normalization to 7000 reads per sample, 36 pairs of BA–BAL samples deriving from 18 patients remained analysable.

### Bacterial Richness and Diversity in Post-LT Airway Samples

**FIGURE 2:**
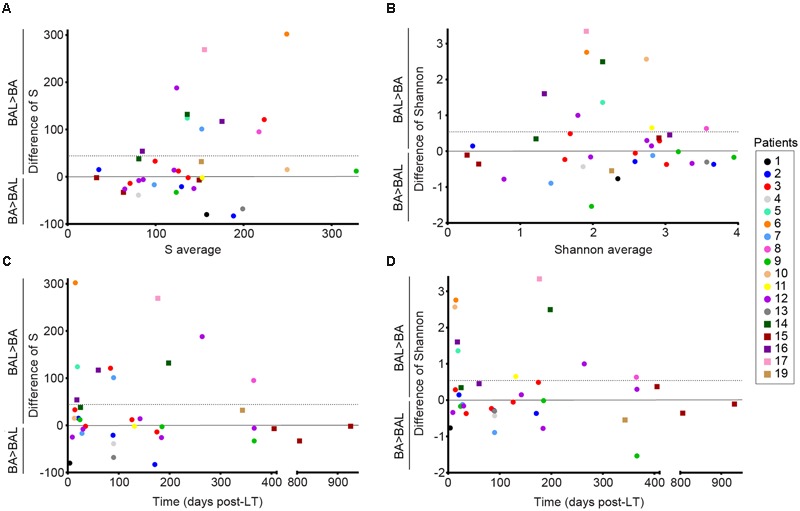
**Alpha-diversity of the conducting and respiratory microbiota of lung-transplanted patients.** Richness was calculated with the S observed estimator, and alpha-diversity was estimated with the Shannon index. Dots represent non-CF patients, whereas squares represent CF-patients. *Y*-axis corresponds to the differences of values obtained for BAL minus the one calculated for BA. The horizontal line represents the mean difference in S or Shannon (BAL-BA). **(A,B)** Bland–Altman plots representing the average of the values obtained for BAL and BA for each pair in X-axis. **(C,D)** Differences of S and Shannon over time post-LT.

We characterized the BA–BAL pairs by calculating the observed OTU richness (S) and the Shannon diversity index. An important variability of both indices was observed between all samples. When all 36 pairs of samples were included, our analysis revealed that the observed richness in BAL samples was in average 44.1 units higher than in BA samples (95% CI 1.8–86.4) (**Figure 2A**). The mean difference was significantly different from 0 (*p*-value = 0.041, linear mixed model). Difference in 95% of the measures ranged between -80 and 269, indicating an important variability. The *S* value decreased by 22% for each year post-LT (**Figure 2C**, Supplementary Table S2). However, statistical analysis on *S* values demonstrated that the effect of time was not significant neither for BA nor for BALs samples (*p* = 0.08, linear regression with mixed effects). No time effect was also reported during the 1st year post-LT (*p* = 0.70). In addition, the interaction term between the time post-LT and the type of sample was not statistically significant (*p* = 0.27).

In addition, we observed that the mean difference in Shannon of BAL minus BA was 0.54 (95%CI -0.01–1.09) (**Figure 2B**). However, the mean difference was not significantly different from 0 (*p*-value = 0.055, linear mixed model). Differences in Shannon values also showed an important variability, with a difference in 95% of the measures comprised between -0.89 and 2.76. The Shannon index showed a decrease of 0.29 units of Shannon for each year post-LT (**Figure 2D**, Supplementary Table S2). However, statistical analysis on Shannon values indicated no significant effect of time neither for BA samples nor BALs (*p* = 0.21, linear regression with mixed effects). No effect of time was also reported during the 1st year post-LT (*p* = 0.46). The interaction term between the time post-LT and the type of sample was not statistically significant (*p* = 0.27).

**FIGURE 3:**
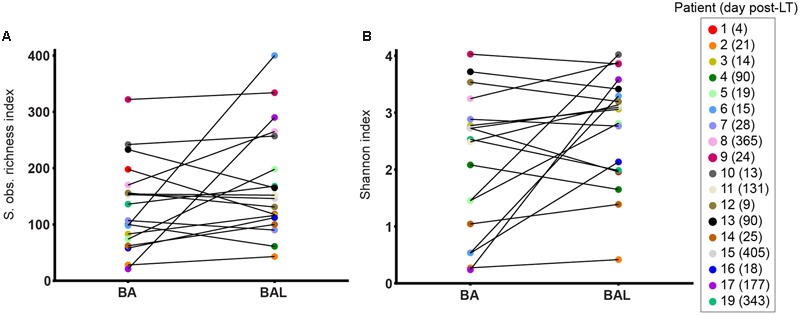
**Alpha-diversity of BA–BAL pairs collected the 1st month post-LT.** S observed richness estimator **(A)** and Shannon index **(B)** calculated for the first BA and BAL pairs collected in 18 patients. The patient’s number is indicated with the day of sampling expressed in day post-LT.

To remove repeated measurements, we analysed only the first BA–BAL pair collected from each of 18 patients. A higher OTU richness was observed in BALs relative to matched BAs (median 438, IQR 198–764 vs. median 396, IQR 264–585, *p* = 1 by Wilcoxon signed-rank test) (**Figure 3**). Likewise, Shannon diversity index was higher in BAL than in BA samples (median 3.09 IQR 2.08–3.40 vs. 2.51 IQR 1.16–2.86, *p* = 0.11, Wilcoxon signed-rank test). However, these differences did not reach the statistical significance.

Distance-based linear model test showed that the variation on Shannon diversity was best explained by the number of antibiotics used (15.6% of the variation, Pseudo-*F* = 6.2771, *p* = 0.018), whereas the time interval after transplantation (3.7% of the variation, Pseudo-*F* = 1.293, *p* = 0.245) and age (4.9% of the variation, Pseudo-*F* = 1.7583, *p* = 0.215) had no effect.

### Bacterial Community Profiles of the Conducting and Respiratory Zones of the Lung Allograft

**FIGURE 4:**
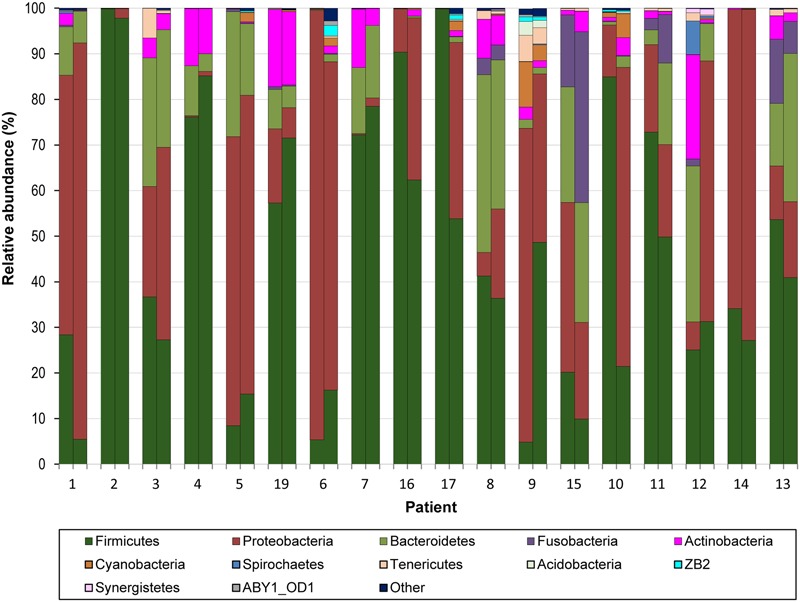
**Relative abundance of bacterial phyla across 18 pairs of BA–BAL samples.** The proportion of 16S sequences assigned to phyla with a relative abundance >1% in at least one sample is presented. Samples are presented by pairs: left, BA; right: BAL.

We mapped the 16S rDNA reads onto the Greengenes database with a 97% identity level and examined bacterial communities at different taxonomic levels. BA and BAL samples were dominated by phyla Firmicutes and Proteobacteria, followed by Bacteroidetes and Actinobacteria (**Figure 4**). These four phyla, identified in all samples, were the only phyla with a median relative abundance >1% in any of the two sample types. We found an inverse correlation between Proteobacteria and Firmicutes (Spearman *r* = –0.830, Supplementary Figure S1). Reads assigned to the phylum Tenericutes were identified in nearly all (35/36) samples but with a low relative abundance (median 0.12 and 0.16% for BA and BAL samples, respectively). Genera *Staphylococcus, Pseudomonas, Streptococcus*, and *Prevotella* were most abundant across samples (Supplementary Figure S2). The proportion of several bacterial taxa differed between BA and BAL samples (Supplementary Table S3) but most of them had low relative abundance. For instance, at the genus and OTU levels, significant changes were observed only for the taxa with a proportion <0.22% of the total bacterial population, except for the two OTUs assigned to *Staphylococcus* (0.41 and 0.52%, respectively).

### Microbiota Clustering

**FIGURE 5:**
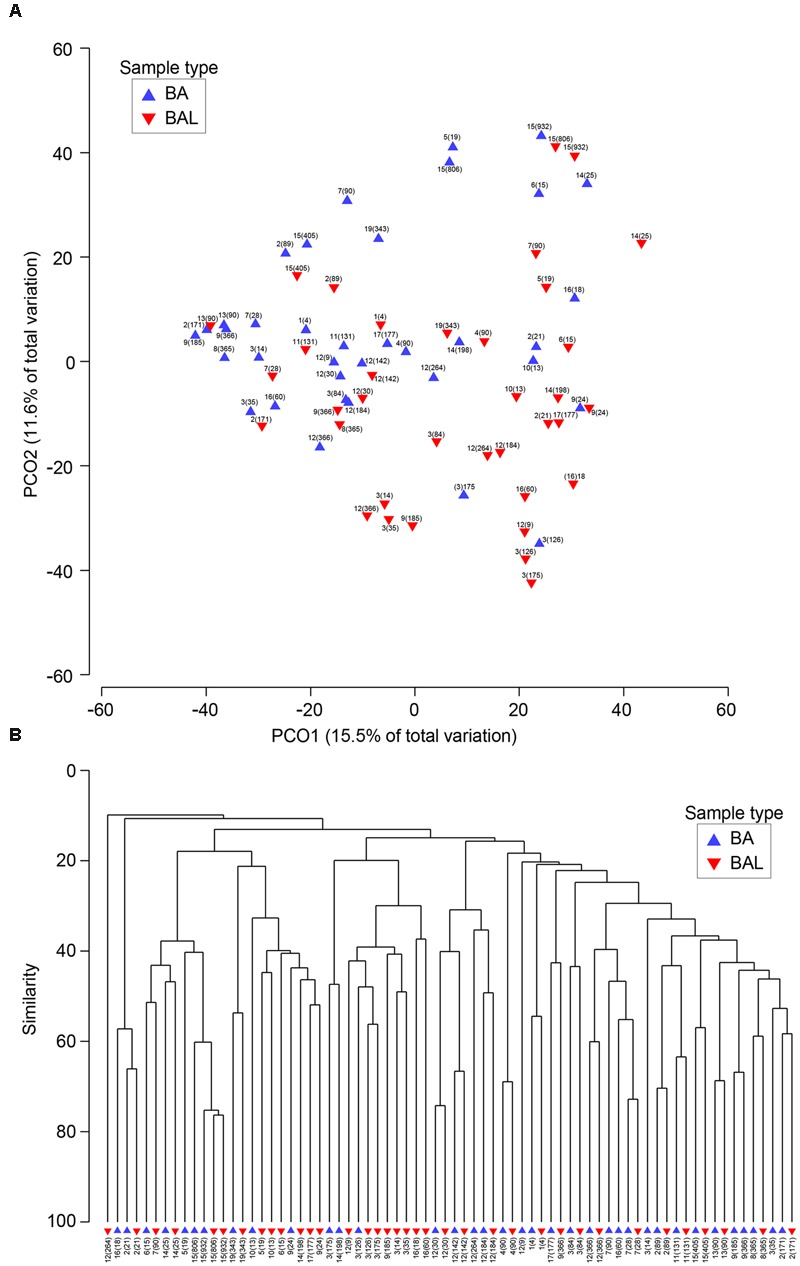
**Similarities between BA and BAL microbiota. (A)** Principal coordinates analysis of 38 BA and BAL pairs. The analysis was done in PRIMER, based on square-root-transformed abundance of operational taxonomic units (OTUs) and Bray–Curtis similarity matrix. **(B)** Group average clustering from Bray–Curtis similarities on root-transformed relative abundances. BAs are represented by a blue triangle whereas BALs are indicated by an inverse red triangle. Each sample is characterized by the patient’s number followed by the day of sampling post-LT in brackets.

We investigated the relationship between respiratory bacterial communities in BAs and BALs. Principal Coordinate analysis and Hierarchical Cluster analysis of all samples showed that BA and BAL of the same pair (same patient and day) clustered together (**Figure 5**). Also, samples from the same individual obtained at different time points tended to form clusters although some of them clustered apart. In the first two principal coordinates, most BAL samples were displaced rightward (30/36), downward (33/36), or both (27/36) relative to the paired BA samples. This distribution suggests a small effect of the sample type on microbiota clustering.

**FIGURE 6:**
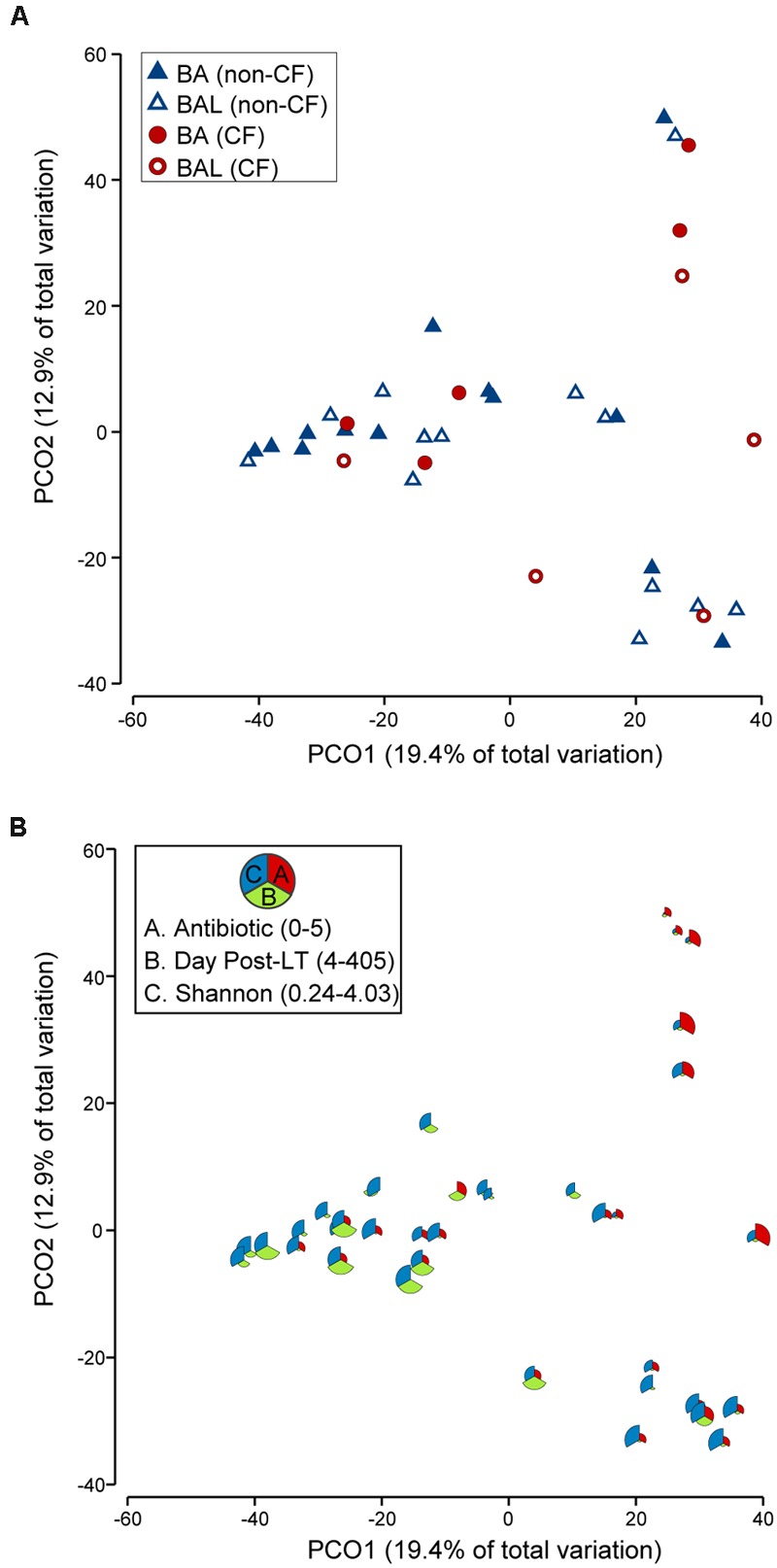
**PCoA of 18 pairs of BA–BAL samples.** Only the first BA–BAL pair from each individual is presented (sampling range: days 4–405, median 24.5 days). The analysis was based on Bray–Curtis similarity matrix obtained on square-root transformed relative abundance of OTUs. **(A)** Symbols indicate the type of sample (BA, BAL) and CF pathology (CF, non-CF). **(B)** This bubble plot depicts the relationship between bacterial community profiles and the number of antibiotics used, day post-LT and Shannon diversity index. The larger the part of the pie is, the higher is the value (antibiotic: 0–5; day post-LT: 4–405; Shannon: 0.24–4.03).

**FIGURE 7:**
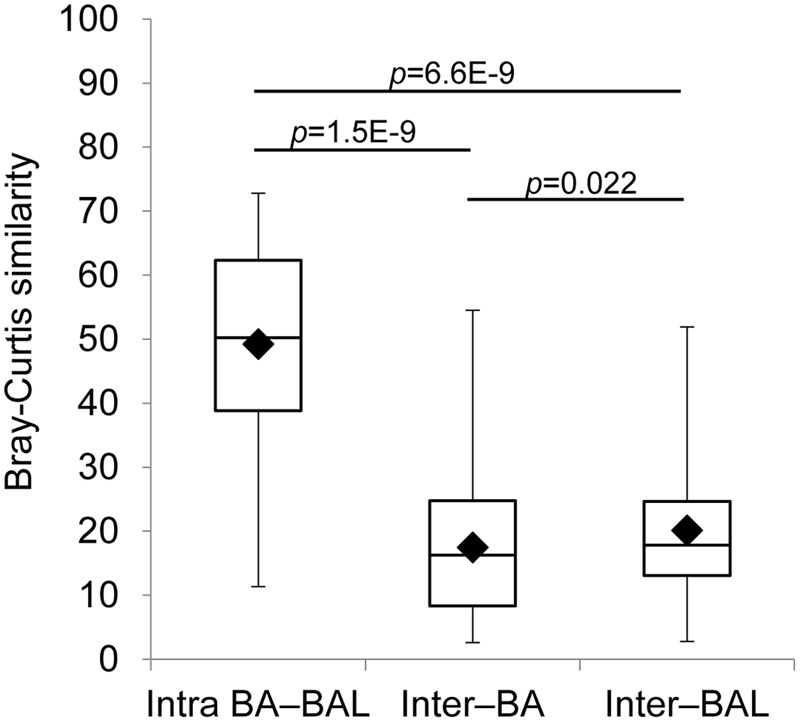
**Intra-individual and inter-individual microbiota similarity.** Only the first BA–BAL pair from each (18) individual is presented. Bray–Curtis similarity was based on square-root transformed relative abundance of OTUs. Intra BA–BAL designate the similarity between BA and BAL samples taken from the same individual at the same sampling point. Inter–BA and Inter–BAL correspond to inter-individual similarity of BA and BAL samples, respectively.

The analysis which excluded repeated measurements of the same individual and included only the first BA–BAL pair from each of 18 patients (**Figure 6**, Supplementary Table S4), confirmed strong clustering by individual (Permanova global test, Pseudo-*F* = 3.8652, *p* = 0.0001). No effect of the type of sample on microbiota profiles was found (Permanova Pseudo-*F* = 1.4323, *p* = 0.1098). The similarity between BAs and BALs microbiota collected at the same sampling point of the same individual was significantly greater than inter-individual similarity observed for each sample type (**Figure 7**). Samples collected from CF patients did not cluster together (**Figure 6A**), suggesting that there was no typical microbiota related to CF-transplanted patients (Permanova Pseudo-*F* = 1.2564, *p* = 0.1836). The relationship between microbiota clustering in PCoA and the Shannon diversity, number of antibiotics used, and time post-transplantation are given in **Figure 6B**.

Distance-based linear model test showed that the variation in microbiota structure was explained by the number of antibiotics used (7.3% of the variation, Pseudo-*F* = 2.685, *p* = 0.0007), and by the time interval after transplantation (6.1% of the variation, Pseudo-*F* = 2.1961, *p* = 0.0095), whereas age had no significant effect (3.9% of the variation, Pseudo-*F* = 1.3821, *p* = 0.14).

The OTUs making the greatest contribution to the microbiota difference between BA and BAL samples were determined using the SIMPER (PRIMER) analysis (Supplementary Figure S3). The two most discriminating OTUs (>2% contribution, square-root transformed data) were OTU139321 from *Pseudomonas* and OTU92651 from *Staphylococcus*. However, the contributions of these OTUs to BALs–BAs dissimilarity were not consistent (dissimilarity/SD of 0.68 and 0.56, respectively), which was also the case with other OTUs.

Microbiota comparisons based on the proportion of genera also showed clustering by individual (Supplementary Figure S2). Again, statistical tests did not find a significant effect of the sample type (Permanova Pseudo-*F* = 1.4283, *p* = 0.1595), CF pathology (Permanova Pseudo-*F* = 1.2094, *p* = 0.2603), or age (DISTLM Pseudo-*F* = 1.6647, *p* = 0.0964) on the microbiota profiles. Variation in community structure was explained by inter-individual differences (global Permanova test Pseudo-*F* = 4.7571, *p* = 0.0001), number of antibiotics administered (DISTLM Pseudo-*F* = 4.252, *p* = 0.0002) and the time interval post-transplantation (DISTLM Pseudo-*F* = 2.5786, *p* = 0.0136).

### Microbiota of the Conducting and Respiratory Zones Display Similar Changes after Transplantation

**FIGURE 8:**
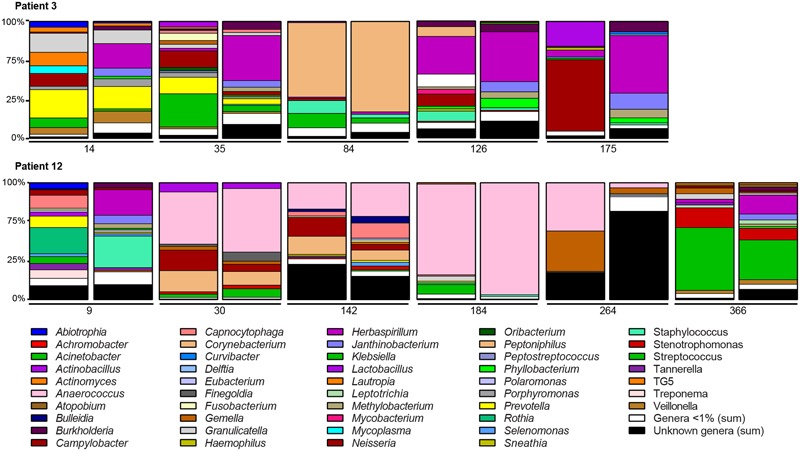
**Longitudinal analysis of the microbiota colonizing the conducting and respiratory zones of two non-CF patients.** Bar graphs indicate the relative abundances of the genera identified by 16S rRNA gene sequencing. Bar graphs are presented by pairs: left, bacterial composition of BAs; right, bacterial composition of BALs. The number below the bar graphs indicates the time of sampling expressed in day post-LT. Only genera with relative abundance of >1% in at least one of the samples are presented. OTUs unassigned to a genus are pooled to represent the category “Unknown genera” while low-abundance genera (<1%) are pooled to represent the category “Genera <1%.”

We investigated changes in the microbiota in five and six BA–BAL pairs collected longitudinally in two patients during 6 months and 1 year post-LT, respectively (**Figure 8**). Longitudinal analysis of BA–BAL pairs of patients #3 and #12 showed dynamic modifications of the microbiota over time. However, the microbiota remained highly similar between pairs of BA and BAL samples. Microbiota of patient #3 was dominated by *Prevotella* at day 14, which was replaced by *Corynebacterium* at day 84. In contrast, *Anaerococcus* was over-represented in BA–BAL pairs of patient #12. The longitudinal microbial colonization of patients #3 and #12 shows a wave-like profile constituted by a dominant species at mid-term. However, the analysis of only two colonization profiles did not allow us to obtain a statistical significance for the time-dependent changes of the microbiota.

## Discussion

In this study, we characterized and compared the microbiota between the conducting (BA) and respiratory zones (BAL) of lung-transplant recipients over time. We show that although bacterial richness was higher in samples from the respiratory as compared to the conducting zone, population diversity and structure between these two sites were similar. Furthermore, we show dynamic changes of the microbiota of both sites over time.

In healthy patients Dickson et al. (2015a) showed decreasing community richness with increasing distance from the source community of the upper respiratory tract. Our finding that, in average, the bacterial richness is higher in respiratory than in conducting airways could be a characteristic of lung-transplant recipients. We could hypothesize that the microbiota of the conducting zone is more representative of the recipient’s microbiota, whereas the microbiota of respiratory airways more reflects the donor microbiota, explaining a higher richness in the respiratory airway samples. Another hypothesis could be that the immunosuppression of lung transplant recipients is responsible for difference between these patients and healthy controls.

Transplanted and non-transplanted patients share a common background lung microbiota, characterized by bacteria found also in the oral and upper respiratory tract (Willner et al., 2013; Becker et al., 2014). Considerable regional variations impacting on bacterial growth and survival exist in lungs, including oxygen tension, temperature, abundance of inflammatory cells, and gastroesophageal reflux (Dickson et al., 2015b). Strikingly in previous reports BALs more closely related to the oral microbiota of the same patient than to BALs of other patients (3). However, the divergence between BALs and oral microbiota was more pronounced in transplant patients than in non-transplant control subjects. Furthermore, oral and proximal lung microbiota of a young CF child were different from the microbiota of distal lung sections (Brown et al., 2014). In the present study lung microbiota post-LT showed a relative spatial homogeneity reflected by similar microbial populations in the BAs and BALs. Given the absence of detectable cross-contamination during an *ex vivo* performed control experiment, it is unlikely that relevant cross-contamination between BA and BAL samples explains this observation.

The intra-patient clustering of BA–BAL pairs from the same day, together with a tendency to cluster for samples at different time-points for each patient contrasts with a lower inter-individual similarity inside a collection zone, and supports an individual microbial signature following LT. CF patients are frequently colonized by bacterial pathogens which can seed the allograft from the nasopharyngeal reservoir (Leung et al., 2008). However, we did not find a typical microbiota CF signature compared to non-CF lung transplant recipients.

We found in both conductive and respiratory zones a large predominance of Firmicutes and Proteobacteria, as well as an inverse correlation between these two phyla. Overrepresentation of Proteobacteria, which includes major lung pathogens, and such an inverse correlation have been previously linked to the severity of disease in COPD patients (Garcia-Nunez et al., 2014). Whether overrepresentation of Proteobacteria in lung transplant recipients is associated with allograft failure remains to be studied in a larger cohort.

While the Bray–Curtis similarity between BA and BAL in pairs of samples did not show any obvious trends in changes over time, variation in microbiota structure was linked to the number of antibiotics used and the time interval after LT. In addition, the antibiotic number and time post-transplantation influenced the dynamic changes of the community structure in both zones over time, confirming previous observations on the impact of antibiotics in CF lung transplant recipients (Willner et al., 2013). Interestingly whereas antibiotics impacted on community structure and diversity, this effect did not endure in non-transplanted CF patients leading to the conclusion that the microbiota is resilient to antibiotic exposure (Fodor et al., 2012; Stressmann et al., 2012; Zhao et al., 2012).

Our study represents the real-life situation with its associated potential limitations. All our patients were exposed to antibiotics impacting both structure and changes of the microbiota. Given the difficulty to obtain clinical samples the number of subjects analyzed is also relatively low precluding a definite conclusion about taxa that may discriminate BA and BAL samples, as well as CF from non-CF recipients.

A relative similarity in microbial profiles in BA and BAL samples may have important clinical implications. BA samples are obtained by less invasive methods than BAL samples and therefore may possibly be used instead of BALs in clinical microbiological diagnostics. In future work, it will be interesting to analyze longitudinal samples collected in pairs from the sinuses, the oral cavity, the main bronchia, and the lower airways. This should provide information on the dynamics of bacterial graft colonization, the contribution of the upper airways in this process and on the microbial movements between the different airway zones.

## Author Contributions

MB, TK, JS, and CvD designed the research; J-DA, OM, and PG collected samples and clinical data; MB performed experiments; MB, VL, NG, LB, LF, TK, and CvD analyzed data; MB, VL, and CvD wrote the paper.

## Conflict of Interest Statement

The authors declare that the research was conducted in the absence of any commercial or financial relationships that could be construed as a potential conflict of interest.

## Footnotes

1 http://vegan.r-forge.r-project.org/
