# Supplementary material for: Microbial Communities of Conducting and Respiratory Zones of Lung-Transplanted Patients
Source: Front Microbiol. 2016 Nov 7;7:1749. doi: 10.3389/fmicb.2016.01749 (PMC5097918; doi:10.3389/fmicb.2016.01749)
Supplement: Supplementary file 1 [file Data_Sheet_1.DOCX]

Supplementary Material

**Microbial communities of conducting and respiratory zones of lung-transplanted patients**

M. Beaume, V. Lazarevic, T. Köhler, N. Gaia, O. Manuel, JD. Aubert, L. Baerlocher, L. Farinelli, P. Gasche, J. Schrenzel, C. van Delden^*^ and the Swiss Transplant Cohort Study

*** Correspondence:** Christian van Delden: christian.vandelden@hcuge.ch

**Supplementary Material**

**gDNA extraction**

One mL of aspiration or lavage samples was treated with 100 µg/mL of dithiothreitol (DTT) and incubated 10 min at 37°C with shaking (220 rpm) to dissociate the mucus. A 500 µL aliquot was frozen and thawed 3 times, and centrifuged for 10 min at 10,000 g (4°C). Chemical and mechanical lysis was performed by adding 180 µL of ATL buffer (DNeasy, QIAGEN) to 250 µL of the supernatants. This mix was further disrupted in a FastPrep instrument (MP Biomedicals) for 4 x 1 min at 6.5M/S with glass beads (acid-washed G1152, Sigma). After removal of the beads by centrifugation (30 s; 10,000 g), gDNA was extracted from supernatants by using the DNeasy kit (QIAGEN) according to the manufacturer’s instructions.

**16S rRNA gene amplification, sequencing and taxonomic analysis**

The hypervariable regions V4-V6 of the 16S rRNA gene were amplified by PCR using primers Uni16_518F (CCAGCAGCYGCGGTAAN) and Uni16_1064R (CGACRRCCATGCANCACCT) (9). The master mix contained 1X Platinum polymerase buffer, 0.8 units PCR Platinum Taq DNA polymerase high fidelity (Invitrogen), 3.7 mM MgSO4, 200 mM dNTPs (each), and 0.4 µM of each primer. Four µL of each total gDNA were directly added to reach a final volume of 50 µL. PCR cycling conditions included initial denaturation at 94°C for 3 min; 30 cycles of 94°C for 30 s, 57°C for 45 s, and 72°C for 1 min; and a final extension at 72°C for 2 min. Quality of PCR amplicons was evaluated on 1% agarose gel. Three replicate reactions were performed and pooled before PCR product purification. PCR products were purified by using the GeneJet PCR purification kit (ThermoScientific) following manufacturer’s recommendations and eluted in 40 µL of 0.1X elution buffer.

**Evaluation of potential cross-contamination between BA and BAL**

Bacterial DNA was extracted from 1 mL of the pseudo-BA and pseudo-BAL. The 1 mL aliquot was frozen and thawed 3 times, and centrifuged for 10 min at 10,000 g (4°C). Chemical and mechanical lysis was performed by adding 180 µL of ATL buffer (DNeasy, QIAGEN) to 250 µL of the supernatants. This mix was further disrupted in a FastPrep instrument (MP Biomedicals) for 4 x 1 min at 6.5M/S with glass beads (acid-washed G1152, Sigma). After removal of the beads by centrifugation (30 s; 10,000 g), gDNA was extracted from supernatants by using the DNeasy kit (QIAGEN) according to the manufacturer’s instructions.

qPCR assay was performed on an RotorGene RG3000 (Corbett Research). Reaction mixtures contained 1× RotorGene SYBR Green PCR Master Mix (QIAGEN), 0.6 μM forward (5’-GCAAGCGCATGGTCGACAAGA-3’) and reverse (5’-CGCTGTGCTCTTGCAGGTTGTGA-3’) *rpsL* primers and 3 μl of DNA extract, in a final volume of 15 µL. Cycling conditions included initial denaturation of 5 min at 95 °C followed by 45 cycles of 95 °C for 5 s and 60° for 10 s. No-template qPCR controls were performed using 3 μl ddH2O instead of DNA extract. The reference curves for DNA quantitation were obtained using known concentrations of genomic DNA of *P. aeruginosa* PA14. All reactions were carried out in duplicate.

**Supplementary Tables**

**Supplementary Table 1. Number of reads analysed, taxonomic richness and diversity indices.**

|  |  |  | Counts (reads) | | S observed | | Shannon | | HAK number** | |
| --- | --- | --- | --- | --- | --- | --- | --- | --- | --- | --- |
| Patient n° | Time post-LT (days) | BA-BAL pair n° | BA | BAL | BA | BAL | BA | BAL | BA | BAL |
| 1 | 4 | 1 | 86400 | 349160 | 198 | 118 | 2,7278 | 1,9596 | HAK-81 | HAK-82 |
| 2 | 21 | 2 | 188277 | 71096 | 28 | 43 | 0,2735 | 0,4175 | HAK-68 | HAK-214 |
| 2 | 89 | 3 | 213936 | 278046 | 140 | 119 | 2,7251 | 2,4352 | HAK-70 | HAK-69 |
| 2 | 171 | 4 | 169655 | 19421 | 230 | 147 | 3,8478 | 3,4811 | HAK-71 | HAK-215 |
| 3 | 14 | 5 | 59002 | 21788 | 83 | 116 | 2,7742 | 3,0611 | HAK-196 | HAK-195 |
| 3 | 35 | 6 | 47801 | 11803 | 138 | 136 | 3,1993 | 2,8299 | HAK-198 | HAK-197 |
| 3 | 84 | 7 | 43782 | 54322 | 163 | 284 | 1,7310 | 1,4978 | HAK-200 | HAK-199 |
| 3 | 126 | 8 | 12754 | 10984 | 120 | 132 | 2,6120 | 2,5572 | HAK-202 | HAK-201 |
| 3 | 175 | 9 | 39462 | 23057 | 78 | 64 | 1,4448 | 1,9332 | HAK-204 | HAK-203 |
| 4 | 90 | 10 | 176423 | 240531 | 100 | 61 | 2,0815 | 1,6500 | HAK-103 | HAK-102 |
| 5 | 19 | 11 | 129451 | 49097 | 74 | 198 | 1,4550 | 2,8151 | HAK-87 | HAK-86 |
| 6 | 15 | 13 | 135317 | 84126 | 98 | 400 | 0,5358 | 3,2928 | HAK-98 | HAK-99 |
| 7 | 28 | 16 | 234597 | 37835 | 107 | 90 | 2,8854 | 2,7644 | HAK-63 | HAK-176 |
| 7 | 90 | 17 | 67408 | 117616 | 102 | 203 | 1,8693 | 0,9763 | HAK-64 | HAK-65 |
| 8 | 365 | 21 | 60747 | 22556 | 170 | 265 | 3,2479 | 3,8802 | HAK-220 | HAK-219 |
| 9 | 24 | 22 | 62122 | 74961 | 322 | 334 | 4,0286 | 3,8575 | HAK-72 | HAK-73 |
| 9 | 185 | 23 | 52728 | 17860 | 154 | 151 | 3,1850 | 3,1706 | HAK-211 | HAK-210 |
| 9 | 366 | 24 | 75023 | 65080 | 140 | 107 | 2,7508 | 1,2118 | HAK-212 | HAK-213 |
| 10 | 13 | 28 | 23746 | 112400 | 242 | 257 | 1,4546 | 4,0211 | HAK-90 | HAK-91 |
| 11 | 131 | 29 | 156763 | 86126 | 154 | 152 | 2,4869 | 3,1382 | HAK-96 | HAK-97 |
| 12 | 9 | 30 | 46621 | 10183 | 156 | 131 | 3,5363 | 3,1960 | HAK-179 | HAK-178 |
| 12 | 30 | 31 | 72147 | 60133 | 85 | 77 | 2,0500 | 1,8886 | HAK-181 | HAK-180 |
| 12 | 142 | 32 | 78373 | 66112 | 114 | 128 | 2,7337 | 2,8815 | HAK-183 | HAK-182 |
| 12 | 184 | 33 | 52477 | 72648 | 78 | 52 | 1,1667 | 0,3857 | HAK-185 | HAK-184 |
| 12 | 264 | 34 | 57194 | 53223 | 30 | 218 | 1,2947 | 2,2931 | HAK-187 | HAK-186 |
| 12 | 366 | 35 | 10551 | 9278 | 89 | 83 | 2,5948 | 2,8926 | HAK-189 | HAK-188 |
| 13 | 90 | 38 | 746584 | 475447 | 233 | 165 | 3,7200 | 3,4163 | HAK-79 | HAK-80 |
| 14 | 25 | 36 | 153186 | 97664 | 62 | 100 | 1,0447 | 1,3888 | HAK-18 | HAK-19 |
| 14 | 198 | 37 | 297403 | 148192 | 70 | 202 | 0,8880 | 3,3824 | HAK-21 | HAK-22 |
| 15 | 405 | 25 | 350792 | 1235360 | 153 | 146 | 2,7245 | 3,0957 | HAK-33 | HAK-34 |
| 15 | 806 | 26 | 20462 | 29018 | 80 | 47 | 0,6076 | 0,2493 | HAK-133 | HAK-134 |
| 15 | 932 | 27 | 15901 | 32373 | 34 | 32 | 0,3219 | 0,2136 | HAK-136 | HAK-135 |
| 16 | 18 | 18 | 61465 | 24027 | 58 | 112 | 0,5313 | 2,1348 | HAK-150 | HAK-151 |
| 16 | 60 | 19 | 44041 | 28596 | 117 | 234 | 2,8322 | 3,2850 | HAK-153 | HAK-152 |
| 17 | 177 | 20 | 130852 | 93628 | 21 | 290 | 0,2394 | 3,5824 | HAK-45 | HAK-46 |
| 18 | 800 | 14 | 1378 | 7598 | nd* | 77 | nd* | 1,8319 | HAK-140 | HAK-139 |
| 18 | 828 | 15 | 16410 | 3747 | 133 | nd* | 3,1392 | nd* | HAK-142 | HAK-141 |
| 19 | 343 | 12 | 325165 | 265245 | 136 | 168 | 2,5317 | 1,9854 | HAK-27 | HAK-28 |

* Alpha-diversity indexes were not calculated due to low sequencing coverage

** Sequence files were deposited in EMBL-ENA under the accession number PRJEB13210.

**Supplementary Table 2. Variations of S and Shannon indexes over time.**

|  | **All follow-up** | | **First year** | |
| --- | --- | --- | --- | --- |
| **S. observed** |  |  |  |  |
|  | RGM* (95%CI) | *p*-value | RGM* (95%CI) | *p*-value |
| Time post-LT (per year) | 0.78 (0.60 to 1.02) | 0.0797 | 1.12 (0.63 to 2.01) | 0.6980 |
| **Shannon** |  |  |  |  |
|  | Mean difference (95%CI) | *p*-value | Mean difference (95%CI) | *p*-value |
| Time post-LT (per year) | -0.29 (-0.73 to 0.16) | 0.2088 | 0.37 (-0.61 to 1.36) | 0.4625 |

* RGM: ratio of geometric mean

Method: Linear regression with mixed effects on intercept and slope of Time post LT to account for repetition of measures.

**Supplementary Table 3. Taxa differentially represented in BA and BAL.**

| Rank | Taxonomic information (phylum;class;order;family;genus;OTU) | Median | | p-value |
| --- | --- | --- | --- | --- |
|  |  | BA | BAL |  |
| Class | Proteobacteria;Alphaproteobacteria | 0.0624 | 1.4642 | 0.0393 |
|  | Cyanobacteria;Chloroplast | 0.0090 | 0.1657 | 0.0393 |
| Order | Proteobacteria;Alphaproteobacteria;Sphingomonadales | 0.0041 | 0.2487 | 0.0266 |
|  | Proteobacteria;Betaproteobacteria;Burkholderiales | 0.1969 | 3.1564 | 0.0266 |
|  | Proteobacteria;Gammaproteobacteria;Enterobacteriales | 0.0175 | 0.1682 | 0.0328 |
| Family | Actinobacteria;Actinobacteria;Actinomycetales;Propionibacteriaceae | 0.0007 | 0.0057 | 0.0409 |
|  | Actinobacteria;Actinobacteria;Coriobacteriales;Coriobacteriaceae | 0.0304 | 0.0008 | 0.0409 |
|  | Chloroflexi;Anaerolineae;Caldilineales;Caldilineaceae | 0.0026 | 0.0016 | 0.0409 |
|  | Firmicutes;Clostridia;Clostridiales;ClostridialesFamilyXIII.IncertaeSedis | 0.0050 | 0.0000 | 0.0413 |
|  | Proteobacteria;Alphaproteobacteria;Rhizobiales;Bradyrhizobiaceae | 0.0002 | 0.0013 | 0.0409 |
|  | Proteobacteria;Alphaproteobacteria;Rhizobiales;Methylobacteriaceae | 0.0022 | 0.1702 | 0.0003 |
|  | Proteobacteria;Alphaproteobacteria;Rhizobiales;Phyllobacteriaceae | 0.0000 | 0.0099 | 0.0225 |
|  | Proteobacteria;Alphaproteobacteria;Rhodobacterales;Rhodobacteraceae | 0.0017 | 0.0110 | 0.0413 |
|  | Proteobacteria;Alphaproteobacteria;Sphingomonadales;Sphingomonadaceae | 0.0038 | 0.2066 | 0.0147 |
|  | Proteobacteria;Betaproteobacteria;Burkholderiales;Comamonadaceae | 0.0272 | 0.7433 | 0.0098 |
|  | Proteobacteria;Betaproteobacteria;Burkholderiales;Oxalobacteraceae | 0.0024 | 0.6288 | 0.0003 |
|  | Proteobacteria;Epsilonproteobacteria;Campylobacterales;Helicobacteraceae | 0.0000 | 0.0012 | 0.0413 |
|  | Proteobacteria;Gammaproteobacteria;Enterobacteriales;Enterobacteriaceae | 0.0175 | 0.1682 | 0.0225 |
|  | Proteobacteria;Gammaproteobacteria;Pseudomonadales;Moraxellaceae | 0.0406 | 1.2615 | 0.0234 |
| Genus | Actinobacteria;Actinobacteria;Actinomycetales;Propionibacteriaceae;  Propionibacterium | 0.0004 | 0.0057 | 0.0248 |
|  | Actinobacteria;Actinobacteria;Coriobacteriales;Coriobacteriaceae;Atopobium | 0.0076 | 0.0002 | 0.0287 |
|  | Bacteroidetes;Flavobacteria;Flavobacteriales;Flavobacteriaceae;Flavobacterium | 0.0001 | 0.0036 | 0.0248 |
|  | Chloroflexi;Anaerolineae;Caldilineales;Caldilineaceae;Caldilinea | 0.0026 | 0.0007 | 0.0248 |
|  | Firmicutes;Clostridia;Clostridiales;ClostridialesFamilyXIII.IncertaeSedis;  Mogibacterium | 0.0018 | 0.0000 | 0.0248 |
|  | Proteobacteria;Alphaproteobacteria;Rhizobiales;Methylobacteriaceae;  Methylobacterium | 0.0022 | 0.1702 | 0.0006 |
|  | Proteobacteria;Alphaproteobacteria;Rhizobiales;Phyllobacteriaceae;  Phyllobacterium | 0.0000 | 0.0098 | 0.0248 |
|  | Proteobacteria;Alphaproteobacteria;Rhodobacterales;Rhodobacteraceae;  Paracoccus | 0.0006 | 0.0049 | 0.0435 |
|  | Proteobacteria;Alphaproteobacteria;Sphingomonadales;Sphingomonadaceae;  Novosphingobium | 0.0001 | 0.0041 | 0.0248 |
|  | Proteobacteria;Alphaproteobacteria;Sphingomonadales;Sphingomonadaceae;  Sphingobium | 0.0017 | 0.0268 | 0.0322 |
|  | Proteobacteria;Alphaproteobacteria;Sphingomonadales;Sphingomonadaceae;  Sphingomonas | 0.0011 | 0.1445 | 0.0139 |
|  | Proteobacteria;Betaproteobacteria;Burkholderiales;Alcaligenaceae;  Azohydromonas | 0.0000 | 0.0009 | 0.0248 |
|  | Proteobacteria;Betaproteobacteria;Burkholderiales;Burkholderiaceae;  Burkholderia | 0.0010 | 0.1209 | 0.0070 |
|  | Proteobacteria;Betaproteobacteria;Burkholderiales;Comamonadaceae;  Acidovorax | 0.0000 | 0.0067 | 0.0480 |
|  | Proteobacteria;Betaproteobacteria;Burkholderiales;Comamonadaceae;  Comamonas | 0.0010 | 0.0147 | 0.0139 |
|  | Proteobacteria;Betaproteobacteria;Burkholderiales;Comamonadaceae;  Curvibacter | 0.0013 | 0.0347 | 0.0336 |
|  | Proteobacteria;Betaproteobacteria;Burkholderiales;Comamonadaceae;Delftia | 0.0000 | 0.0080 | 0.0287 |
|  | Proteobacteria;Betaproteobacteria;Burkholderiales;Comamonadaceae;  Rhodoferax | 0.0000 | 0.0012 | 0.0287 |
|  | Proteobacteria;Betaproteobacteria;Burkholderiales;Oxalobacteraceae;  Herbaspirillum | 0.0000 | 0.1459 | 0.0287 |
|  | Proteobacteria;Betaproteobacteria;Burkholderiales;Oxalobacteraceae;  Janthinobacterium | 0.0010 | 0.1324 | 0.0070 |
|  | Proteobacteria;Gammaproteobacteria;Pseudomonadales;Moraxellaceae;  Acinetobacter | 0.0139 | 0.2113 | 0.0248 |
| OTU | Actinobacteria;Actinobacteria;Actinomycetales;Propionibacteriaceae;  Propionibacterium;Propionibacteriumacnes;OTU368907 | 0.0000 | 0.0036 | 0.0372 |
|  | Actinobacteria;Actinobacteria;Coriobacteriales;Coriobacteriaceae;Atopobium;;  OTU529659 | 0.0066 | 0.0000 | 0.0372 |
|  | Firmicutes;Bacilli;Bacillales;Staphylococcaceae;Staphylococcus;;OTU323445 | 0.0000 | 0.0201 | 0.0358 |
|  | Firmicutes;Bacilli;Bacillales;Staphylococcaceae;Staphylococcus;;OTU519827 | 0.0099 | 0.5242 | 0.0358 |
|  | Firmicutes;Bacilli;Bacillales;Staphylococcaceae;Staphylococcus;  Staphylococcus aureus;OTU217996 | 0.0070 | 0.4071 | 0.0363 |
|  | Firmicutes;Bacilli;Lactobacillales;Lactobacillaceae;Lactobacillus;  Lactobacillus reuteri;OTU262735 | 0.0006 | 0.0000 | 0.0428 |
|  | Firmicutes;Bacilli;Lactobacillales;Streptococcaceae;Streptococcus;;OTU561636 | 0.0594 | 0.0024 | 0.0363 |

Only the first BA–BAL pair from each of 18 individual is presented. Taxa with a median abundance >0 in at least one of the two sample types were analyzed. Only taxa with significant changes (P<0.05 Wilcoxon signed-rank test with Benjamini-Hochberg correction) are presented.

**Supplementary Table 4. Number of antibiotics received at the first sampling date**

| **Patient n°** | Age at LT | First sampling date (days post-LT) | Number of antibiotic received |
| --- | --- | --- | --- |
| **1** | 63 | 4 | 1 |
| **2** | 61 | 21 | 1 |
| **3** | 63 | 14 | 1 |
| **4** | 60 | 90 | 0 |
| **5** | 52 | 19 | 0 |
| **6** | 44 | 15 | 1 |
| **7** | 55 | 28 | 0 |
| **8** | 54 | 365 | 0 |
| **9** | 47 | 24 | 1 |
| **10** | 61 | 13 | 1 |
| **11** | 54 | 131 | 0 |
| **12** | 58 | 9 | 1 |
| **13** | 50 | 90 | 0 |
| **14** | 32 | 25 | 5 |
| **15** | 39 | 405 | 1 |
| **16** | 15 | 18 | 3 |
| **17** | 27 | 177 | 2 |
| **19** | 20 | 343 | 1 |


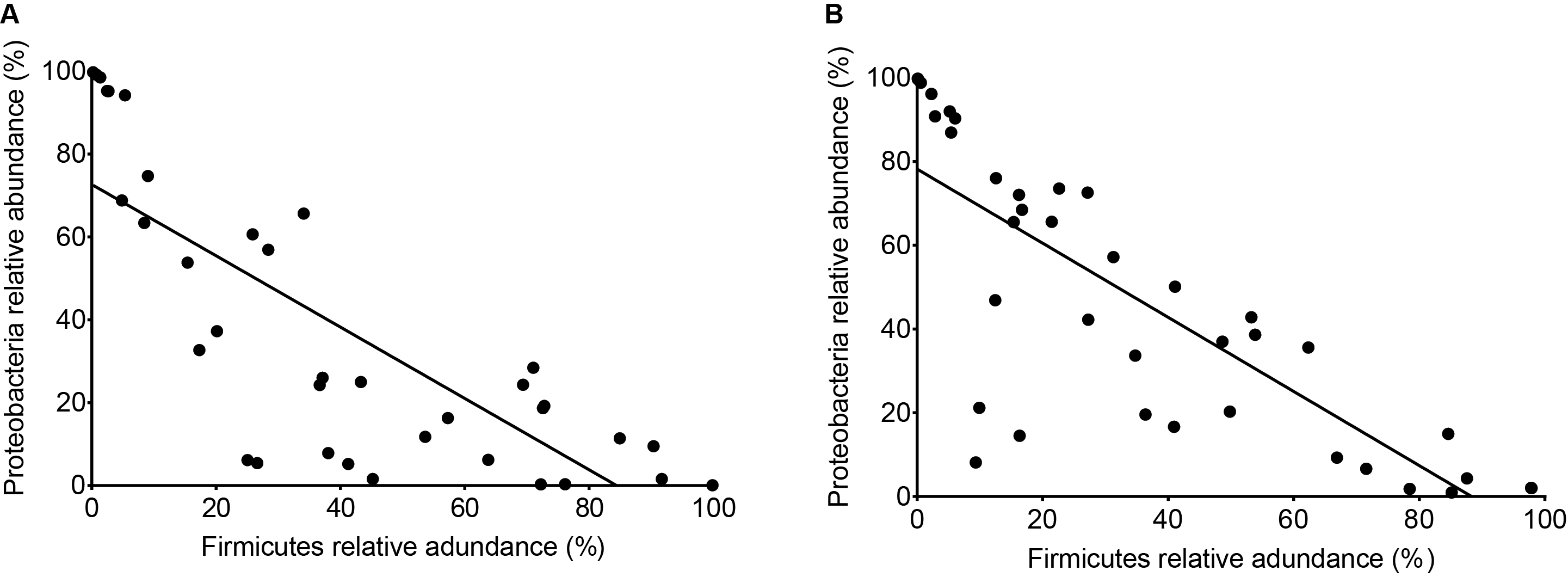


**Supplementary Figure 1. Scatterplots of Proteobacteria and Firmicutes relative abundances in BAs and BALs.** Panel A: BAs; Panel B: BALs.


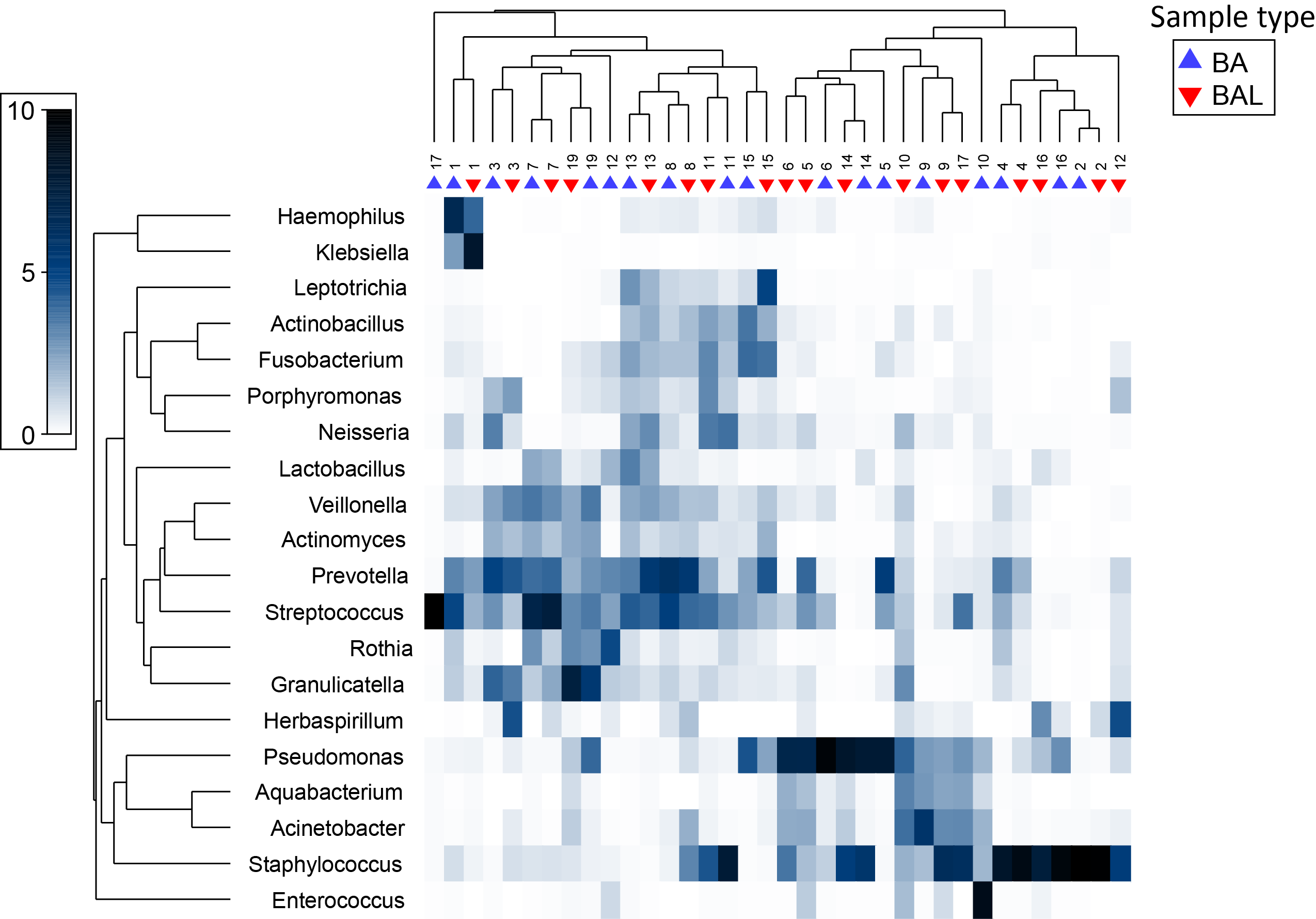


**Supplementary Figure 2. Relative abundance of bacterial genera across 18 pairs of BA–BAL samples.** The proportion of 16S sequences assigned to top 20 genera (mean relative abundance >0.85 %). The relative abundance (square root transformed) is given according to the colour intensity gradient on the left.


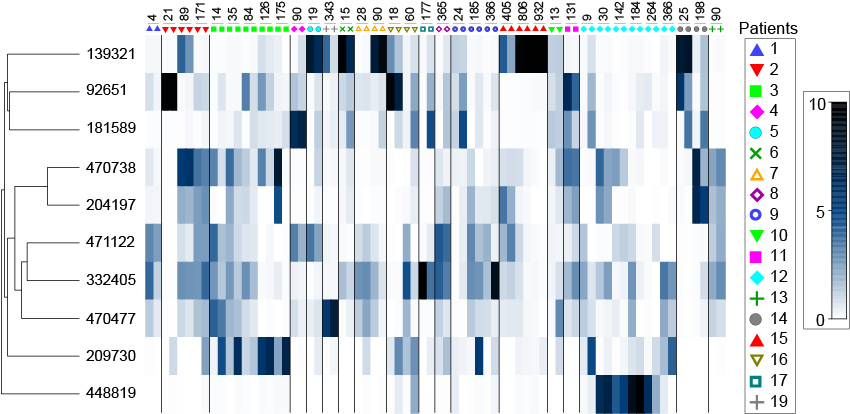


**Supplementary Figure 3. OTUs with the highest contribution to BAL-BA distinction.** Top ten OTUs that make the greatest contribution to the microbiota difference between BA and BAL samples were identified by SIMPER. The relative OTU abundance (square root transformed) is given according to the colour intensity gradient on the right. For the same individual (symbol) and sampling point (day after transplantation indicated above the symbol) left and right bars correspond to BA and BAL, respectively.
